# Patterns of combustible and electronic cigarette use during pregnancy and associated pregnancy outcomes

**DOI:** 10.1038/s41598-021-92930-5

**Published:** 2021-06-29

**Authors:** Annette K. Regan, Gavin Pereira

**Affiliations:** 1 School of Nursing and Health Professions, University of San Francisco, San Francisco, CA USA; 2 UCLA Fielding School of Public Health, Los Angeles, CA USA; 3 School of Public Health, Curtin University, Perth, WA Australia; 4 Telethon Kids Institute, Perth, WA Australia; 5 Centre for Fertility and Health (CeFH), Norwegian Institute of Public Health, Oslo, Norway

**Keywords:** Epidemiology, Medical research, Outcomes research

## Abstract

Although pregnant smokers may perceive electronic cigarettes (e-cigarettes) as safe alternatives to smoking combustible cigarettes, few studies have evaluated perinatal e-cigarette use and its associated health effects. We analyzed data from the Pregnancy Risk Assessment Monitoring System (PRAMS, 2016–2018) for 16,022 women who recently gave birth and reported smoking combustible cigarettes prior to pregnancy. Using average marginal predictive values from multivariable logistic regression to produce adjusted prevalence ratios (aPRs), we estimated the prevalence of combustible cigarette smoking during pregnancy and adverse birth outcomes associated with e-cigarette use. In total, 14.8% of smoking women reported using e-cigarettes prior to pregnancy. There was no association between e-cigarette use prior to pregnancy and combustible cigarette smoking during pregnancy (aPR 0.95; 95% CI 0.88, 1.02); however, e-cigarette use during pregnancy was associated with higher prevalence of combustible cigarette smoking during pregnancy (aPR 1.65; 95% CI 1.52, 1.80). In this sample, we did not observe evidence to support reduced risk of preterm birth, small-for-gestational age and low birthweight compared to combustible cigarette smoking during pregnancy. The prevalence of LBW was higher for those who used e-cigarettes, even exclusively, compared to women who quit smoking cigarettes entirely. These results suggest that e-cigarettes should not be considered a safe alternative to combustible cigarette smoking during pregnancy.

## Introduction

Electronic cigarettes (or e-cigarettes) are electronic nicotine delivery systems which have grown in popularity and use since their introduction to the US in 2007^1,2^. Although there remains limited evidence demonstrating e-cigarettes as an effective smoking cessation method^3–5^, e-cigarettes are currently aggressively marketed toward cigarette smokers as such^6^, and previous studies have shown that cigarette smokers may perceive these products as potential quit aids^7,8^. One population group which may be susceptible to such messaging is pregnant women^9–11^, with some studies suggesting such marketing has led to an increase in use of e-cigarettes among pregnant women^12^. An estimated 7% of women use e-cigarettes around the time of pregnancy, and 45% of e-cigarette users believed e-cigarettes were less harmful than combustible cigarette smoking and may help them quit or reduce combustible cigarette smoking during pregnancy^13^. This use stands in contrast to the US Preventive Services Task Force (USPSTF) statement, asserting there is insufficient evidence to recommend e-cigarettes as a tobacco cessation tool for adults, including nonpregnant and pregnant smokers^14^.

Adverse health effects in humans have been recently documented, linking e-cigarette use with increased risk of lung injury^15–17^. In addition to directly impacting lung health, previous animal studies have shown that offspring from mothers exposed to e-cigarettes can result in neurodevelopmental impairments^18^, diminished lung development^19^, reduced crown-rump length and fetal weight^20^, and increased oxidative stress and inflammation^19,21^. Two recent epidemiological studies in humans have shown that e-cigarette use during pregnancy is associated with increased risk of fetal growth restriction^22,23^ and low birthweight^24^. Despite this evidence, the fetal health impact of e-cigarette use during pregnancy as compared to quitting smoking has not yet been evaluated. Furthermore, given pregnant women and women planning pregnancy may be a particularly sensitive group to e-cigarette advertising, additional epidemiological research evaluating the prevalence and patterns of e-cigarette use among combustible cigarette smokers is warranted.

The aims of the present study were to (1) describe patterns of e-cigarette use among women who smoked combustible cigarettes prior to becoming pregnant; and (2) assess whether e-cigarette use during pregnancy was associated with adverse birth outcomes in comparison to continued combustible cigarette smoking.

## Methods

We analyzed Phase 8 (2016–2018) data from the Pregnancy Risk Assessment Monitoring System (PRAMS). PRAMS is a routine, ongoing surveillance system collecting information on preconception, prenatal and postpartum health which is implemented by states and coordinated by the Centers for Disease Control and Prevention (CDC)^25^. The PRAMS study protocol has been approved by the Institutional Review Boards of CDC and each participating site. Our study proposal was reviewed and approved by the Pregnancy Risk Assessment Monitoring System (PRAMS) Working Group. As part of this surveillance system, a representative sample of 1000–3000 women with a recent live birth is drawn from the state’s birth certificate datafile each year. Selected women are first contacted by mail, and after attempts to contact women by mail, those who do not respond are next contacted by telephone, and up to 15 attempts are made to contact participants by phone. Contact is initiated within two to four months following delivery^25^.

For respondents, questionnaire responses are linked to information extracted from the birth certificate, including sociodemographic and maternal and infant health information. The current study sample was restricted to women with a recent live birth, with a singleton pregnancy with a birthweight ≥ 400 g, who self-reported smoking combustible cigarettes during the two years preceding pregnancy, and had complete exposure, outcome and covariate information.

### Exposure definition

We used questionnaire data from the PRAMS Phase 8 core questionnaire to identify combustible and e-cigarette use prior to and during pregnancy. Women were asked to self-report whether they had smoked combustible cigarettes (yes/no) or used e-cigarettes (yes/no) during the three months prior to pregnancy. They were also asked about their combustible cigarette use and e-cigarette use during the last three months of pregnancy. Women who continued to smoke combustible cigarettes during pregnancy were also asked to report on the number of cigarettes smoked daily: < 1 cigarette, 1–5 cigarettes, 6–10 cigarettes, 11–20 cigarettes, or ≥ 21 cigarettes.

We classified women into four mutually exclusive categories based on these responses: (1) those who quit combustible cigarette smoking prior to pregnancy and did not use e-cigarettes (Former Smokers); (2) those who quit combustible cigarette smoking but used e-cigarettes during pregnancy (E-cigarette only smokers); (3) those who continued smoking combustible cigarettes and also used e-cigarettes during pregnancy (Dual Users); and (4) those who continued smoking combustible cigarettes and did not use e-cigarettes (Current Smokers).

### Outcome definitions

We first considered the prevalence of combustible cigarette smoking by e-cigarette use, which was defined as self-reported use of combustible cigarettes during the last three months of pregnancy. We next considered the prevalence of adverse birth outcomes. Birthweight (in grams), gestational age (in weeks), and an indicator of birthweight in the lowest 10th percentile for gestational age were obtained from the linked birth certificate data. Using these data, we examined three categorical birth outcomes: preterm birth, small-for-gestational-age (SGA), and low birthweight (LBW). Preterm birth was defined based on a clinical estimate of gestational age of < 37 weeks, SGA was defined in the birth certificate as a birthweight in the lowest 10th percentile for gestational age, and low birthweight was defined as an infant with a birthweight < 2500 g.

### Definition of covariates

We used data from the linked birth certificate and PRAMS questionnaire to define covariates of interest that have previously been associated with adverse birth outcomes. Covariates were considered for inclusion in the analysis based on a directed acyclic graph outlining the potential relationship between e-cigarette use and fetal health (Supplemental Figure S1). Possible covariates included maternal age, race/ethnicity, education, marital status, residence, insurance status during pregnancy, adequacy of prenatal care, use of Special Supplement Nutrition Program for Women, Infant and Children (WIC) services, presence of an obstetric risk factor, parity, pregnancy intention, and multivitamin use. Adequacy of prenatal care was assessed using the Adequacy of Prenatal Care Utilization (APNCU) Index, which is derived from birth certificate information on when prenatal care began and the number of prenatal care visits^26^. Multivitamin use was selected as a marker of health-seeking behavior. Obstetric risk factors related to the pregnancy included pre-pregnancy diabetes, gestational diabetes, pre-pregnancy hypertension, gestational hypertension, hypertension eclampsia, previous preterm birth, infertility treatment, use of assisted reproductive technology, and previous cesarean section.

### Statistical analysis

We included data from 38 PRAMS sites which met the CDC threshold for response rate of ≥ 55% between 2016 and 2018, including Alaska, Alabama, Arkansas, Colorado, Connecticut, Delaware, Georgia, Hawaii, Iowa, Illinois, Kansas, Kentucky, Louisiana, Massachusetts, Maryland, Maine, Michigan, Missouri, Montana, North Carolina, North Dakota, Nebraska, New Hampshire, New Jersey, New Mexico, New York (and New York City), Oklahoma, Pennsylvania, Rhode Island, South Dakota, Texas, Utah, Virginia, Washington, Wisconsin, West Virginia, and Wyoming. PRAMS data are weighted to account for nonresponse, noncoverage and complex sampling design^25^. To account for this weighting, analyses were performed using SAS-callable SUDAAN version 11.0.3 (Research Triangle Institute, NC, United States). We estimated weighted percentages and corresponding 95% confidence intervals (CIs) for responses. We compared the frequency of cigarettes smoked during pregnancy for current smokers and dual users using Chi-squared tests.

We used average marginal values from multivariable regression models to calculate adjusted marginal prevalences, unadjusted and adjusted prevalence ratios (aPR) and corresponding 95% CIs of combustible cigarette smoking during pregnancy for those who used e-cigarettes prior to or during pregnancy vs. those who did not. Models adjusted for maternal age, race/ethnicity, education, adequacy of prenatal care, multivitamin use, rurality of residence, WIC access, and pregnancy intention. Sub-analyses considered the frequency of e-cigarette use prior to and during pregnancy.

We developed similar models to compare the prevalence of preterm birth, SGA and LBW by e-cigarette and combustible cigarette use during pregnancy. Using average marginal values from multivariable regression models, we estimated PRs of birth outcomes for dual users, e-cigarette only users, and former smokers as compared to current smokers. Adjustment variables were selected a priori based on a directed acyclic graph (Supplemental Figure S1). The final adjusted model controlled for maternal age, race/ethnicity, parity, adequacy of prenatal care, multivitamin use, and presence of an obstetric risk factor.

### Role of the funding source

Although this work received financial support from the National Health and Medical Research Council (GNT1099655; GNT1173991) and the Research Council of Norway Centres of Excellence (#262700), the funders had no involvement in the research activities, including the study design, data collection or analysis, interpretation of findings, writing of the manuscript, or the decision to publish. The authors had full access to the study data and had the final responsibility for the decision to submit for publication.

## Results

Of 20,547 respondents who self-reported smoking combustible cigarettes in the two years preceding pregnancy, 19,711 respondents had singleton pregnancies with birthweight ≥ 400 g (Supplemental Figure S2); 3689 had any missing information for key analytic variables: 68 were missing birth outcome information; 669 were missing self-reported e-cigarette or combustible cigarette information; and 2952 were missing relevant covariate information (range of missing values: 51 missing parity to 1993 missing maternal race/ethnicity). The final sample for analysis included 16,022 respondents.

### Patterns in combustible and e-cigarette use

**Figure 1:**
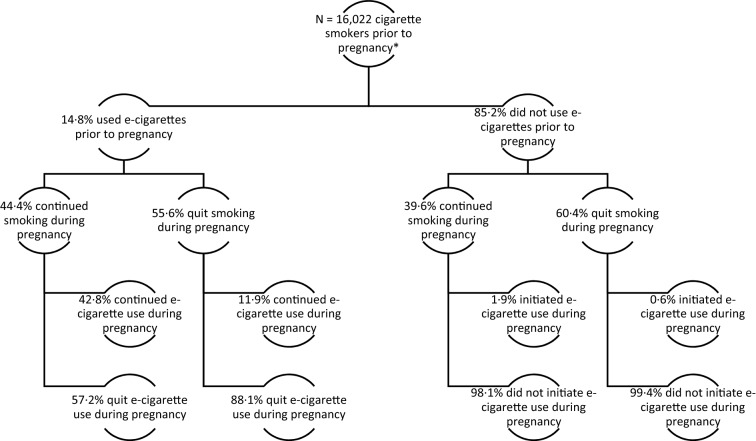
Patterns of e-cigarette use and combustible cigarette smoking prior to and during pregnancy among US women (N = 16,022)—Pregnancy Risk Assessment Monitoring Survey, United States, 2016–2018*. *PRAMS sites included in this analysis are: Alaska (2016-2018), Alabama (2017), Arkansas (2016), Colorado (2016–2018), Connecticut (2016-2018), Delaware (2016-2018), Georgia (2017–2018), Hawaii (2016), Iowa (2016–2017), Illinois (2016–2017), Kansas (2017–2018), Kentucky (2017–2018), Louisiana (2016–2018), Massachusetts (2016–2018), Maryland (2016–2017), Maine (2016–2017), Michigan (2016–2018), Missouri (2016–2018), Montana (2017), North Carolina (2017), North Dakota (2017), Nebraska (2016,2018), New Hampshire (2016–2017), New Jersey (2016–2018), New Mexico (2016–2018), New York (2016–2017), New York City (2016–2018), Oklahoma (2016–2017), Pennsylvania (2016–2018), Rhode Island (2016–2018), South Dakota (2017–2018), Texas (2016), Utah (2016–2018), Virginia (2016–2018), Washington (2016–2018), Wisconsin (2016–2018), West Virginia (2016–2018), and Wyoming (2016–2018). *Women who self-reported smoking combustible cigarettes during the 3 months prior to becoming pregnancy; sample size reflects the unweighted sample size.

Among the 16,022 women who self-reported smoking combustible cigarettes during the three months prior to becoming pregnant, 14.8% (95% CI 13.9, 15.7%) reported also using e-cigarettes prior to pregnancy (Fig. 1). Of these women, 44.4% (95% CI 41.2, 47.6%) continued to smoke combustible cigarettes and 55.6% (95% CI 52.4, 58.8%) quit combustible cigarettes during pregnancy. Nearly half (42.8%; 95% CI 38.1, 47.6%) of e-cigarette users who continued smoking combustible cigarettes during pregnancy also continued e-cigarette use during pregnancy. Among the 85.2% (95% CI 84.3, 86.1%) of combustible cigarette smokers who did not use e-cigarettes prior to pregnancy, 60.4% (95% CI 59.1, 61.7%) quit smoking during pregnancy. Few women who quit combustible cigarette use during pregnancy and did not use e-cigarettes prior to pregnancy initiated e-cigarette use during pregnancy (0.6%; 95% CI 0.4, 0.8%). The prevalence of pregnant women with a history of combustible cigarette smoking and who used e-cigarettes during pregnancy ranged from 3.7% (95% CI 3.9, 5.6%) in 2016 to 4.9% (95% CI 3.0, 6.1%) in 2018.

Overall, we observed no difference in the prevalence of combustible cigarette smoking among women who used e-cigarettes prior to pregnancy compared to women who did not (aPR 0.95; 95% CI 0.88, 1.02) (Table 1). However, women who used e-cigarettes less than daily during the three months prior to pregnancy had a higher prevalence of combustible cigarette smoking during pregnancy compared to women who used e-cigarettes daily (aPR 1.35; 95% CI 1.16, 1.56). The prevalence of combustible cigarette smoking was higher among women who used e-cigarettes during pregnancy compared to non-users (aPR 1.65; 95% CI 1.52, 1.80), and women who used e-cigarettes less than daily during the last three months of pregnancy had a higher prevalence of combustible cigarette smoking compared to women who used e-cigarettes daily (aPR 1.31; 95% CI 1.14, 1.50). We found no difference in the number of cigarettes smoked for women used e-cigarettes during (*P* = 0.15) as compared to smokers who did not use e-cigarettes (Supplemental Figure S3).

**Table 1:** Prevalence of combustible cigarette smoking by electronic cigarette use prior to and during pregnancy and US women (N = 16,022)—Pregnancy Risk Assessment Monitoring Survey, United States, 2016–2018.

Electronic cigarette (e-cigarette) use	Unadjusted prevalence	Adjusted prevalence	PR (95% CI)	aPR (95% CI)
**Used e-cigarettes prior to pregnancy**				
Yes	44.4 (41.2, 47.6)	42.1 (39.2, 45.0)	**0**.**89 (0**.**82, 0**.**96)**	0.95 (0.88, 1.02)
No	39.6 (38.3, 40.9)	40.0 (38.7, 41.3)	Reference	Reference
**Frequency of use prior to pregnancy**				
Less than daily	49.4 (45.3, 53.4)	49.1 (45.2, 53.0)	**1**.**36 (1**.**16, 1**.**60)**	**1**.**35 (1**.**16, 1**.**56)**
Daily	36.3 (31.4, 41.5)	36.5 (31.8, 41.4)	Reference	Reference
**Used e-cigarettes during pregnancy**				
Yes	73.2 (68.0, 77.9)	64.6 (59.2, 69.7)	**1**.**89 (1**.**76, 2**.**04)**	**1**.**65 (1**.**52, 1**.**80)**
No	38.6 (37.4, 39.9)	39.1 (37.9, 40.3)	Reference	Reference
**Frequency of use during pregnancy**				
Less than daily	81.5 (75.2, 86.5)	74.0 (67.0, 80.0)	**1**.**33 (1**.**14, 1**.**55)**	**1**.**31 (1**.**14, 1**.**50)**
Daily	61.3 (52.6, 69.4)	52.8 (44.8, 60.7)	Reference	Reference

Bolded text indicates significant at p < 0.05.

Adjusted by maternal age, race/ethnicity, and education, adequacy of prenatal care, multivitamin use, rurality of residence, WIC access, and pregnancy intention.

PRAMS sites included in this analysis are: Alaska (2016–2018), Alabama (2017), Arkansas (2016), Colorado (2016–2018), Connecticut (2016–2018), Delaware (2016–2018), Georgia (2017–2018), Hawaii (2016), Iowa (2016–2017), Illinois (2016–2017), Kansas (2017–2018), Kentucky (2017–2018), Louisiana (2016–2018), Massachusetts (2016–2018), Maryland (2016–2017), Maine (2016–2017), Michigan (2016–2018), Missouri (2016–2018), Montana (2017), North Carolina (2017), North Dakota (2017), Nebraska (2016, 2018), New Hampshire (2016–2017), New Jersey (2016–2018), New Mexico (2016–2018), New York (2016–2017), New York City (2016–2018), Oklahoma (2016–2017), Pennsylvania (2016–2018), Rhode Island (2016–2018), South Dakota (2017–2018), Texas (2016), Utah (2016–2018), Virginia (2016–2018), Washington (2016–2018), Wisconsin (2016–2018), West Virginia (2016–2018), and Wyoming (2016–2018).

Based on this information, we classed 8938 women as former smokers (58.5%; 95% CI 57.3, 59.7%), 189 as e-cigarette only users (1.3%; 95% CI 1.0, 1.6%), 585 as dual users (3.4%; 95% CI 3.0, 3.9%), and 6310 as current smokers (36.8%; 95% CI 35.7, 38.0%) (Table 2). Compared to former smokers, a higher percentage of respondents who used e-cigarettes during pregnancy were 18–24 years of age, non-Hispanic white, had less than or equal to 12 years of education, had public health insurance, accessed WIC during pregnancy, had inadequate prenatal care, resided in rural areas of the US, and were combustible cigarette smokers (Table 2). A lower percentage of e-cigarette users reported using a multivitamin and were married.

**Table 2:** Characteristics of respondents, by electronic cigarette (e-cigarette) use during the last 3 months of pregnancy—Pregnancy Risk Assessment Monitoring Survey,* United States, 2016–2018 (n = 16,022).

Characteristic	Former smokers (unweighted n = 8938)	E-cigarette only users (unweighted n = 189)	Dual users (unweighted n = 585)	Current smokers (unweighted n = 6310)
	Weighted % (95% CI)^†^	Weighted % (95% CI)^†^	Weighted % (95% CI)^†^	Weighted % (95% CI)^†^
**Total**	58.5 (57.3, 59.7)	1.3 (1.0, 1.6)	3.4 (3.0, 3.9)	36.8 (35.7, 38.0)
**Maternal age**^**§**^				
18–24 years	30.9 (29.4, 32.4)	41.3 (31.2, 52.2)	31.8 (26.0, 38.3)	30.8 (28.9, 32.6)
25–29 years	31.6 (30.1, 33.1)	24.6 (16.6, 34.8)	35.3 (29.4, 41.8)	34.7 (32.9, 36.7)
30–34 years	24.5 (23.1, 25.9)	19.0 (12.4, 27.9)	23.8 (18.8, 29.7)	22.5 (20.9, 24.2)
35–39 years	10.7 (9.9, 11.7)	15.0 (7.8, 26.7)	8.6 (5.7, 12.9)	10.0 (8.9, 11.2)
≥ 40 years	2.3 (1.8, 2.8)	0.2 (0.0, 1.4)	0.4 (0.2, 0.8)	2.0 (1.5, 2.6)
**Maternal race/ethnicity**^**§**^				
White, non-Hispanic	68.2 (66.7, 69.7)	79.9 (70.2, 87.1)	90.7 (87.2, 93.2)	75.4 (73.7, 77.0)
Black, non-Hispanic	14.8 (13.7, 16.0)	7.9 (3.8, 15.9)	5.7 (3.6, 8.7)	16.6 (15.2, 18.1)
Hispanic	14.8 (13.7, 16.0)	10.8 (5.8, 19.2)	3.3 (2.0, 5.3)	6.6 (5.7, 7.6)
Asian, non-Hispanic	1.7 (1.3, 2.1)	0.3 (0.1, 1.3)	0.3 (0.1, 1.4)	0.8 (0.5, 1.0)
Other, non-Hispanic	0.6 (0.4, 0.9)	1.0 (0.1, 6.9)	0.1 (0.0, 0.3)	0.7 (0.5, 1.1)
**Married**^**§**^	46.4 (44.8, 48.0)	34.4 (24.8, 45.5)	28.6 (23.1, 34.7)	28.0 (26.3, 29.8)
**Maternal education**^**§**^				
< 12 years	11.3 (10.3, 12.4)	17.8 (10.7, 28.1)	25.4 (19.8, 31.8)	22.9 (21.3, 24.6)
12 years	32.1 (30.6, 33.7)	29.1 (21.0, 38.8)	42.0 (35.7, 48.5)	40.2 (38.3, 42.2)
13–15 years	34.4 (32.9, 36.0)	43.3 (32.9, 54.3)	29.8 (24.5, 35.7)	32.4 (30.5, 34.3)
≥ 16 years	22.1 (20.8, 23.5)	9.8 (4.5, 20.0)	2.9 (1.4, 6.1)	4.5 (3.8, 5.3)
**Rural residence**^**§**^	17.1 (15.9, 18.2)	19.7 (13.0, 28.6)	30.0 (24.4, 36.2)	27.0 (25.3, 28.7)
**Insurance used for prenatal care**^**§**^				
Private	35.9 (34.4, 37.5)	22.0 (14.4, 32.0)	12.8 (8.9, 18.1)	13.8 (12.5, 15.3)
Public	58.2 (56.5, 59.8)	73.2 (62.2, 82.0)	82.4 (76.8, 86.9)	79.8 (78.1, 81.4)
Other	4.5 (3.8, 5.3)	4.8 (1.2, 16.7)	4.4 (2.4, 7.8)	5.4 (4.5, 6.5)
None	1.4 (1.0, 2.0)	0	0.4 (0.2, 0.8)	0.9 (0.6 ,1.4)
**Accessed WIC during pregnancy**^**§**^	42.5 (40.9, 44.1)	47.1 (36.6, 57.9)	61.6 (55.0, 67.8)	60.0 (58.1, 62.0)
**Any obstetric risk factor identified**^**¶**^	20.2 (19.0, 21.4)	19.0 (12.6, 27.7)	13.2 (9.7, 17.8)	20.8 (19.3, 22.4)
Parity**				
Primiparous	44.7 (43.1, 46.3)	39.3 (29.4, 50.1)	31.8 (25.8, 38.4)	26.3 (24.6, 28.0)
1 prior birth	31.6 (30.1, 33.1)	30.3 (21.4, 41.0)	27.9 (22.7, 33.8)	32.5 (30.6, 34.4)
2 prior births	14.6 (13.5, 15.8)	20.2 (12.7, 30.5)	21.8 (16.9, 27.6)	21.5 (19.9, 23.1)
≥ 3 prior births	9.1 (8.3, 10.1)	10.3 (4.9, 20.2)	18.5 (14.1, 24.0)	19.8 (18.2, 21.5)
**Intended pregnancy**^**§**^	50.2 (48.5, 51.8)	37.2 (27.4, 48.3)	34.2 (28.3, 40.7)	36.2 (34.3, 38.1)
**First prenatal care (PNC) visit in first trimester**^**§**^	96.0 (95.4, 96.6)	94.8 (90.2, 97.4)	91.2, (87.1, 94.1)	92.4 (91.3, 93.4)
**Adequacy of PNC**^**††**^				
Adequate plus	33.6 (32.2, 35.2)	27.7 (19.4, 38.1)	25.5 (20.3, 31.3)	29.9 (28.1, 31.7)
Adequate	43.8 (42.2, 45.4)	50.8 (40.1, 61.5)	36.7 (30.7, 43.2)	38.8 (36.9, 40.8)
Intermediate	10.5 (9.6, 11.5)	8.7 (4.2, 17.2)	10.5 (7.1, 15.4)	11.4 (10.2, 12.7)
Inadequate	12.0 (11.0, 13.1)	12.8 (8.0, 19.7)	27.3 (21.8, 33.5)	19.9 (18.3, 21.5)
**Multivitamin use**^**§§**^	35.7 (34.2, 37.2)	26.1 (17.5, 36.9)	24.9 (19.9, 30.6)	25.6 (23.9, 27.4)
**Frequency of e-cigarette use**^**§**^				
Daily	–	59.3 (48.4, 69.3)	34.4 (28.6, 40.7)	–
Less than daily	–	40.7 (30.7, 51.6)	65.6 (59.3, 71.4)	–

WIC, the Special Supplemental Nutrition Program for Women, Infant sand Children; PNC, prenatal care.

*PRAMS sites included in this analysis are: Alaska (2016–2018), Alabama (2017), Arkansas (2016), Colorado (2016–2018), Connecticut (2016–2018), Delaware (2016–2018), Georgia (2017–2018), Hawaii (2016), Iowa (2016–2017), Illinois (2016–2017), Kansas (2017–2018), Kentucky (2017–2018), Louisiana (2016–2018), Massachusetts (2016–2018), Maryland (2016–2017), Maine (2016–2017), Michigan (2016–2018), Missouri (2016–2018), Montana (2017), North Carolina (2017), North Dakota (2017), Nebraska (2016, 2018), New Hampshire (2016–2017), New Jersey (2016–2018), New Mexico (2016–2018), New York (2016–2017), New York City (2016–2018), Oklahoma (2016–2017), Pennsylvania (2016–2018), Rhode Island (2016–2018), South Dakota (2017–2018), Texas (2016), Utah (2016–2018), Virginia (2016–2018), Washington (2016–2018), Wisconsin (2016–2018), West Virginia (2016–2018), and Wyoming (2016–2018).

^†^Weighted percentage and corresponding 95% confidence intervals; overall percentages reflect row percentages and percentages by characteristics reflect column percentages.

^§^Significant at *P* < 0.001.

^¶^ Significant at *P* < 0.05.

**Significant at *P* < 0.01.

^‡^Presence of an obstetric risk factor included pre-pregnancy diabetes, gestational diabetes, pre-pregnancy hypertension, gestational hypertension, hypertension eclampsia, previous preterm birth, infertility treatment, use of assisted reproductive technology, and previous cesarean section.

^††^Adequacy of prenatal care was assessed using the Adequacy of Prenatal Care Utilization (APNCU) Index, derived from birth certification information on when prenatal care began and the number of prenatal care visits; Significant at *P* < 0.001.

^§§^Self-reported use of a multivitamin at least once per week; Significant at *P* < 0.001.

### Association with birth outcomes

Among all respondents, 8.9% (95% CI 8.4, 9.5%) of births were preterm, 13.7% (95% CI 12.9, 14.6%) were SGA, and 8.4% (95% CI 8.0, 8.9%) were LBW. Compared to current smokers, women who quit smoking combustible cigarettes and did not use e-cigarettes (former smokers) had a lower prevalence of preterm birth (aPR 0.70; 95% CI 0.61, 0.81), SGA (aPR 0.46; 95% CI 0.41, 0.53) and LBW (aPR 0.53; 95% CI 0.47, 0.60) (Table 3). While the prevalence of adverse birth outcomes appeared to be lower among e-cigarette only users compared to current smokers, there was no significant difference in the prevalence of preterm birth (aPR 0.85; 95% CI 0.55, 1.31), SGA (aPR 0.56; 95% CI 0.29, 1.08), or LBW (aPR 0.81; 95% CI 0.54, 1.21) for e-cigarette users compared to current smokers. When we compared e-cigarette users to former smokers, e-cigarette only users and dual users had similar prevalences of preterm birth (aPR 1.21; 0.78, 1.87 and aPR 1.26; 95% CI 0.91, 1.73, respectively). However, compared to former smokers, e-cigarette only users had a higher prevalence of LBW (aPR 1.52; 95% CI 1.01, 2.29), and dual users had a higher prevalence of both LBW (aPR 2.11; 95% CI 1.6, 2.77) and SGA (aPR 2.60; 95% CI 2.00, 3.38). E-cigarette only users had a similar prevalence of SGA birth compared to former smokers (aPR 1.22; 0.63, 2.34). Similarly, we did not observe any differences in the prevalence of adverse birth outcomes for dual users compared to current smokers.

**Table 3:** Prevalence of select birth outcomes by self-reported electronic cigarette (e-cigarette) use and combustible cigarette smoking during the last 3 months of pregnancy among US women (N = 16,022)—Pregnancy Risk Assessment Monitoring Survey,* United States, 2016–2018.

Birth outcome	Former smokers (n = 8938)	E-cigarette only users (n = 189)	Dual users (n = 585)	Current smokers (n = 6310)
**Preterm birth**				
Unadjusted prevalence (%, 95% CI)^†^	7.7 (7.0, 8.4)	8.5 (5.5, 13.0)	8.3 (6.0, 11.4)	11.0 (10.0, 12.0)
Adjusted prevalence (%, 95% CI)^§^	7.6 (6.9, 8.3)	9.2 (5.9,13.9)	9.6 (7.0, 12.9)	10.8 (9.8, 11.9)
Unadjusted PR (95% CI)^¶^	**0.70 (0.61, 0.80)**	0.78 (0.50, 1.21)	0.76 (0.54, 1.06)	Reference
Adjusted PR (95% CI)^¶^	**0.70 (0.61, 0.81)**	0.85 (0.55, 1.31)	0.88 (0.64, 1.22)	Reference
**Small-for-gestational age**				
Unadjusted prevalence (%, 95% CI)^†^	9.4 (8.5, 10.3)	11.5 (5.9, 21.1)	24.5 (19.2, 30.7)	19.7 (18.2, 21.3)
Adjusted prevalence (%, 95% CI)^§^	9.3 (8.4, 10.2)	11.3 (5.8, 20.9)	24.1 (18.8, 30.4)	20.0 (18.5, 21.7)
Unadjusted PR (95% CI)^¶^	**0.48 (0.42, 0.54)**	0.58 (0.30, 1.11)	1.24 (0.97, 1.59)	Reference
Adjusted PR (95% CI)^¶^	**0.46 (0.41, 0.53)**	0.56 (0.29, 1.08)	1.20 (0.94, 1.55)	Reference
**Low birthweight**				
Unadjusted prevalence (%, 95% CI)^†^	6.3 (5.8, 6.8)	8.8 (5.8, 13.0)	11.3 (8.6, 14.6)	11.5 (10.7, 12.4)
Adjusted prevalence (%, 95% CI)^§^	6.2 (5.7, 6.7)	9.4 (6.3, 13.9)	13.0 (10.0, 16.8)	11.7 (10.8, 12.6)
Unadjusted PR (95% CI)^¶^	**0.55 (0.49, 0.61)**	0.76 (0.50, 1.15)	0.98 (0.74, 1.29)	Reference
Adjusted PR (95% CI)^¶^	**0.53 (0.47, 0.60)**	0.81 (0.54, 1.21)	1.12 (0.85, 1.46)	Reference

Bolded text indicates significant at p < 0.05.

*PRAMS sites included in this analysis are: Alaska (2016–2018), Alabama (2017), Arkansas (2016), Colorado (2016–2018), Connecticut (2016–2018), Delaware (2016–2018), Georgia (2017–2018), Hawaii (2016), Iowa (2016–2017), Illinois (2016–2017), Kansas (2017–2018), Kentucky (2017–2018), Louisiana (2016–2018), Massachusetts (2016–2018), Maryland (2016–2017), Maine (2016–2017), Michigan (2016–2018), Missouri (2016–2018), Montana (2017), North Carolina (2017), North Dakota (2017), Nebraska (2016, 2018), New Hampshire (2016–2017), New Jersey (2016–2018), New Mexico (2016–2018), New York (2016–2017), New York City (2016–2018), Oklahoma (2016–2017), Pennsylvania (2016–2018), Rhode Island (2016–2018), South Dakota (2017–2018), Texas (2016), Utah (2016–2018), Virginia (2016–2018), Washington (2016–2018), Wisconsin (2016–2018), West Virginia (2016–2018), and Wyoming (2016–2018).

^†^Unadjusted prevalence estimated from the weighted percentage and corresponding 95% confidence intervals.

^‡^Adjusted prevalence calculated as the average marginal predictive values.

^¶^Unadjusted and adjusted prevalence ratio of birth outcome and corresponding 95% confidence interval. Adjusted prevalence ratios control for by maternal age, race/ethnicity, adequacy of prenatal care (based on the Adequacy of Prenatal Care Utilization (APNCU) Index), parity, multivitamin use, and presence of an obstetric risk factor.

## Discussion

To our knowledge, this is the first study to evaluate patterns in perinatal e-cigarette use and its associated fetal health effects in a large sample of US women who smoked combustible cigarettes prior to pregnancy. Similar to previous studies^22,24^, we found that many e-cigarette users also smoked combustible cigarettes, and there was no indication that e-cigarette use prior to or during pregnancy was associated with a reduction in combustible cigarette smoking. Women who used e-cigarettes prior to pregnancy did not appear to quit smoking combustible cigarettes any more or less than women who did not use e-cigarettes. In fact, women who used e-cigarettes during pregnancy were more likely to smoke and smoked a similar number of cigarettes compared to non-users. The prevalence of preterm birth, SGA and LBW was similar for e-cigarette use as compared to continued combustible cigarette smoking, and e-cigarette use was associated with increased prevalence of LBW in comparison to abstinence from combustible and electronic cigarettes. In combination, these results suggest pregnant women are not using e-cigarettes as a quit aid and there were no observable fetal health advantages of e-cigarette use over quitting smoking.

Few studies have focused on patterns of e-cigarette use prior to and during pregnancy. Based on our findings, there was no indication in our study that e-cigarette use was associated with reductions in combustible cigarette consumption in this population group. Despite the growing evidence evaluating the impact of e-cigarette use on combustible cigarette consumption, there is no clear consensus on the effect of e-cigarettes on combustible cigarette smoking. A recent systematic review by Kalkhoran and Glantz^3^ indicated that e-cigarette use was associated with a 28% reduction in the odds of smoking cessation. However, a recent Cochrane review of 21 cohort studies and three randomized trials indicated there was sufficient evidence from randomized trials to indicate a beneficial effect of e-cigarettes^4,5^. In two clinical trials, e-cigarette use resulted in lower rates of smoking after six months of follow-up and fewer cigarettes smoked compared with placebo; however, there was no benefit when compared to nicotine replacement therapy^4,5^. Additional studies which evaluate the effect of e-cigarettes specifically among pregnant smokers would be helpful.

We did not observe a significantly lower prevalence of adverse birth outcomes associated with e-cigarette use in comparison to combustible cigarette smoking. However, abstinence from combustible cigarette smoking and e-cigarette use was associated with lower prevalence of preterm birth, SGA and LBW. These findings align with several published animal and human studies that have consistently documented a link between e-cigarette use and markers of fetal growth restriction^20,22,23^. In a mouse study by Orzabal et al.^20^, chronic exposure to e-cigarettes during pregnancy resulted in decreased pup weight body fat, and crown-rump length, which is a measure of fetal growth and a marker of decreased uterine and fetal umbilical blood flow. A recent population-based study of 31,973 new mothers by Wang et al.^22^ observed a two-fold increase in the odds of SGA birth for e-cigarette users compared to non-users. In a prospective cohort study of 232 pregnant women, Cardenas et al.^23^ identified a two- to three-fold increase in the risk of SGA for e-cigarette users compared to non-users.

As e-cigarette products often contain nicotine and are known to result in measurable exposure to nicotine metabolites and total nicotine equivalents^27^, these findings may be explained through prenatal exposure to nicotine. Nicotine is a developmental toxicant^28^, and inhaled nicotine is known to reduce uterine artery blood flow and induce fluctuations in systemic blood pressure^29^. Both of these physiological changes during pregnancy may reduce uteroplacental blood flow resulting in fetal growth restriction and other adverse fetal outcomes. Because studies have shown that these reductions are not observed for nicotine-free e-cigarettes^19^, the current evidence is suggestive that nicotine included in e-cigarettes may adversely influence fetal growth. However, this conclusion cannot be drawn based on the results of the current study or the available evidence, and further research would be needed to confirm this.

It has been well-established that smoking cessation is associated with reduced rates of SGA and other birth outcomes^30^. Given that we did not observe evidence for a reduced prevalence of combustible cigarette smoking or adverse birth outcomes associated with e-cigarette use, the harmful association between e-cigarette use and LBW in comparison to complete abstinence, and the lower prevalence of adverse birth outcomes observed for former smokers, complete cessation of e-cigarette and combustible cigarette use is likely to be optimal for infant health. Women who smoke combustible cigarettes and are planning to become pregnant or are currently pregnant should be counseled on the health risks of smoking either combustible cigarettes or e-cigarette use during pregnancy. Rather than attempting to use e-cigarettes to aid in smoking cessation, healthcare professionals treating pregnant women who smoke should advise them to use evidence-based strategies for cessation, including counseling and use of quitline services^31^.

Our study had several strengths. We included PRAMS data from a large population-based sample of 16,022 women residing in 38 US sites who had a recent live birth and smoked combustible cigarettes prior to becoming pregnant. Linkage of these questionnaire data with birth certificate data allowed us the opportunity evaluate medically-recorded birth outcomes in relation to e-cigarette use. Despite these strengths, the study also had several limitations. First, because PRAMS is an observational, cross-sectional study, we cannot exclude the possibility that our findings may be influenced by residual confounding and other biases, including recall bias, reporting errors, and nondisclosure of substance use during pregnancy. Second, the wording of the PRAMS e-cigarette questionnaire items introduced some restrictions to our analyses. For example, the questionnaire collects data on prenatal use of e-cigarettes only for the last three months of pregnancy, and information on e-cigarette use earlier in pregnancy was not available. Since the majority of women who self-report use of e-cigarettes in the last three months of pregnancy also report using e-cigarettes in the three months prior to becoming pregnant^22^, it is likely that e-cigarette use occurred throughout pregnancy. However, based on the data collected as part of this study, we cannot draw inferences on exposure to e-cigarettes earlier in pregnancy. Third, our analyses were restricted to 16,022 PRAMS respondents who reported smoking prior to becoming pregnant. While this represents a large sample of women with a history of combustible cigarette smoking, this sample size reduced the precision of our estimates. As a result, it is possible that there may be some reduction in the risk of adverse birth outcomes associated with switching to e-cigarette use, and we were not powered to detect this reduction. Based on post hoc power analysis, our study was only powered to detect ± 53% change in the prevalence of preterm birth, ± 31% change in SGA, and ± 51% change in LBW. Despite this, because we observed increased prevalence of LBW among e-cigarette users in comparison to former smokers, abstinence from both combustible and electronic cigarettes was associated with optimal fetal health outcomes. Finally, the PRAMS survey did not provide information for all participating sites on the reasoning for e-cigarette use; therefore, the reasoning for e-cigarette use around the time of pregnancy is unclear in our study. Future studies should consider reasons for e-cigarette use prior to and during pregnancy.

## Conclusions

Among women with a history of combustible cigarette smoking prior to pregnancy, there was no indication that e-cigarette use helped pregnant women to reduce their combustible cigarette consumption or resulted in observable fetal health benefits. These findings support abstinence from both combustible cigarette and e-cigarette use during pregnancy. For women needing support in abstaining from combustible cigarette smoking during pregnancy, healthcare providers should advise women to use evidence-based strategies for promoting smoking cessation during preconception and prenatal care.

## Supplementary Information

**Supplementary Figures**.

## Data Availability

The data used in this study are public use PRAMS multi-state data and are available upon formal request to the CDC (See https://www.cdc.gov/prams/prams-data/researchers.htm).
