# Supplementary material for: Patterns of combustible and electronic cigarette use during pregnancy and associated pregnancy outcomes
Source: Sci Rep. 2021 Jun 29;11:13508. doi: 10.1038/s41598-021-92930-5 (PMC8241992; doi:10.1038/s41598-021-92930-5)
Supplement: Supplementary file 1 — Supplementary Figures. [file 41598_2021_92930_MOESM1_ESM.docx]

**Supplemental Material To:** **Regan AK, Pereira G. Patterns of combustible and electronic cigarette use during pregnancy and associated pregnancy outcomes.**

**
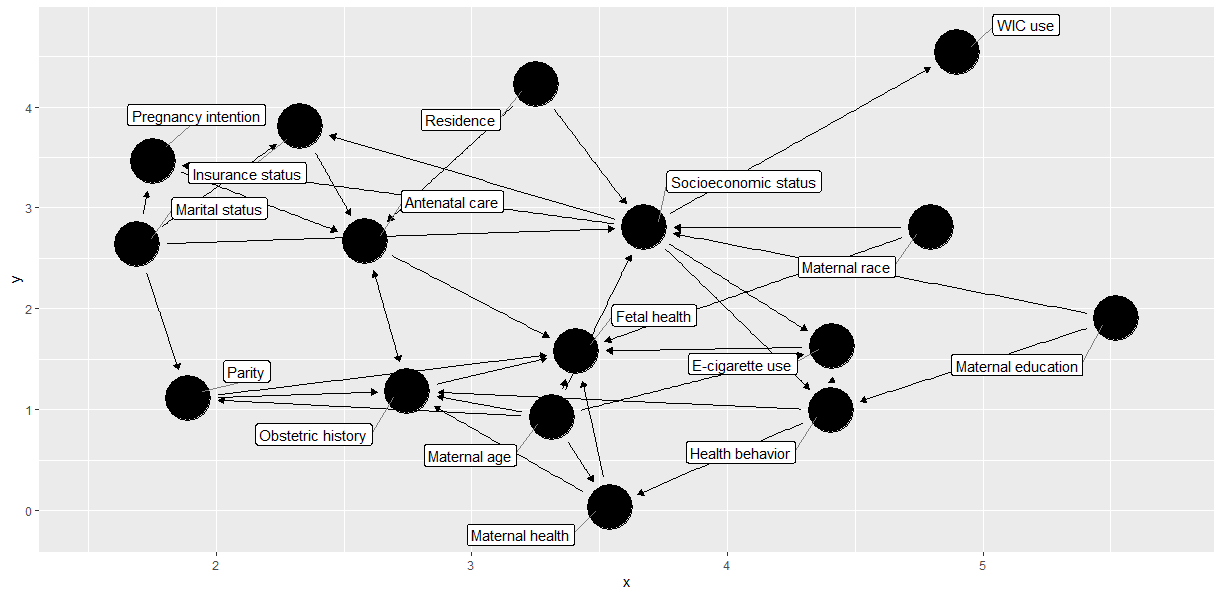
Figure S1.** Directed acyclic graph modeling the relationship between electronic cigarette use and fetal health.

Abbreviations: WIC, Special Supplemental Nutrition Program for Women, Infants, and Children

**Figure S2.** Selection of respondent data for analysis from the Phase-8 Pregnancy Risk Assessment Monitoring System, 38 Sites, United States, 2016-2018.

Respondents who smoked cigarettes in the two years prior to becoming pregnant

(*n* = 20,547)

Respondents with plural births or births <400 grams were excluded (*n* = 836)

Respondents with singleton births and birthweight ≥400 grams (*n* = 19,711)

Respondents with missing data (*n* = 3,491):

- Missing exposure information (*n* = 471)
- Missing outcome information (*n* = 68)
- Missing covariate information (*n* = 2,952)
  - Parity (*n* = 51)
  - Vitamin use (*n* = 78)
  - Obstetric history (*n* = 143)
  - Maternal age (*n* = 229)
  - Adequacy of PNC (*n* = 629)
  - Maternal race (*n* = 1,993)

Respondents included in final analysis (*n* = 16,220)

**Figure S3.** Number of combustible cigarettes smoked each day during the last 3 months of pregnancy, by electronic cigarette (e-cigarette) use during the last 3 months of pregnancy – Pregnancy Risk Assessment Monitoring Survey, United States, 2016-2018.*

*PRAMS sites included in this analysis are: Alaska (2016-2018), Alabama (2017), Arkansas (2016), Colorado (2016-2018), Connecticut (2016-2018), Delaware (2016-2018), Georgia (2017-2018), Hawaii (2016), Iowa (2016-2017), Illinois (2016-2017), Kansas (2017-2018), Kentucky (2017-2018), Louisiana (2016-2018), Massachusetts (2016-2018), Maryland (2016-2017), Maine (2016-2017), Michigan (2016-2018), Missouri (2016-2018), Montana (2017), North Carolina (2017), North Dakota (2017), Nebraska (2016,2018), New Hampshire (2016-2017), New Jersey (2016-2018), New Mexico (2016-2018), New York (2016-2017), New York City (2016-2018), Oklahoma (2016-2017), Pennsylvania (2016-2018), Rhode Island (2016-2018), South Dakota (2017-2018), Texas (2016), Utah (2016-2018), Virginia (2016-2018), Washington (2016-2018), Wisconsin (2016-2018), West Virginia (2016-2018), and Wyoming (2016-2018).
